# Assessment of peritubular capillary rarefaction in kidneys of cats with chronic kidney disease

**DOI:** 10.1111/jvim.16656

**Published:** 2023-02-17

**Authors:** Rene E. Paschall, Jessica M. Quimby, Rachel E. Cianciolo, Shannon M. McLeland, Katharine F. Lunn, Jonathan Elliott

**Affiliations:** ^1^ Department of Clinical Sciences, College of Veterinary Medicine Ohio State University Columbus Ohio USA

**Keywords:** capillary density, CD31, chronic renal disease, feline

## Abstract

**Background:**

Hypoxia is a key driver of fibrosis and is associated with capillary rarefaction in humans.

**Objectives:**

Characterize capillary rarefaction in cats with chronic kidney disease (CKD).

**Animals:**

Archival kidney tissue from 58 cats with CKD, 20 unaffected cats.

**Methods:**

Cross‐sectional study of paraffin‐embedded kidney tissue utilizing CD31 immunohistochemistry to highlight vascular structures. Consecutive high‐power fields from the cortex (10) and corticomedullary junction (5) were digitally photographed. An observer counted and colored the capillary area. Image analysis was used to determine the capillary number, average capillary size, and average percent capillary area in the cortex and corticomedullary junction. Histologic scoring was performed by a pathologist masked to clinical data.

**Results:**

Percent capillary area (cortex) was significantly lower in CKD (median 3.2, range, 0.8‐5.6) compared to unaffected cats (4.4, 1.8‐7.0; *P* = <.001) and was negatively correlated with serum creatinine concentrations (*r* = −.36, *P* = .0013), glomerulosclerosis (*r* = −0.39, *P* = <.001), inflammation (*r* = −.30, *P* = .009), and fibrosis (*r* = −.30, *P* = .007). Capillary size (cortex) was significantly lower in CKD cats (2591 pixels, 1184‐7289) compared to unaffected cats (4523 pixels, 1801‐7618; *P* = <.001) and was negatively correlated with serum creatinine concentrations (*r* = −.40, *P* = <.001), glomerulosclerosis (*r* = −.44, *P* < .001), inflammation (*r* = −.42, *P* = <.001), and fibrosis (*r* = −.38, *P* = <.001).

**Conclusions and Clinical Importance:**

Capillary rarefaction (decrease in capillary size and percent capillary area) is present in kidneys of cats with CKD and is positively correlated with renal dysfunction and histopathologic lesions.

**Abbreviations**
BUN: blood urea nitrogen
CKD: chronic kidney disease
CMJ: corticomedullary junction
HIF: hypoxia‐inducible factor
IRIS: International Renal Interest Society
USG: urine specific gravity
VEGF: vascular endothelial growth factor

## 1 INTRODUCTION

Peritubular capillary rarefaction refers to microvessel loss and decreased microvascular density and is considered an important histologic feature of chronic kidney disease (CKD) in both humans and experimental animal models of kidney injury.^1^, ^2^, ^3^, ^4^ Capillary rarefaction is positively correlated with decreasing kidney function and fibrosis and is considered a major contributor to hypoxia and progression of disease.^1^, ^2^, ^3^, ^4^ The mechanisms by which it occurs are complex and likely involve some combination of pericyte damage and detachment, endothelial cell apoptosis, downregulation of vascular endothelial growth factor (VEGF), upregulation of antiangiogenic factors, and incompetence of endothelial progenitor cells.^1^, ^2^ These processes are likely initiated by damage to tubular epithelial cells and pericytes that result in apoptosis or transdifferentiation into extracellular matrix‐producing myofibroblasts.^2^, ^3^ The resulting interstitial fibrosis broadens the diffusion distance of oxygen between capillaries and tubular epithelial cells, leading to further damage from hypoxia and ischemic injury; the final result is tubular atrophy with loss of nephrons.^2^, ^3^ This loss increases the workload on the remaining nephrons and exacerbates renal hypoxia to which tubular epithelial cells are particularly susceptible.^3^, ^5^

Studies in humans and rats demonstrate that hypoxia is the prominent pathophysiologic process that leads to fibrosis and end‐stage CKD, regardless of the initiating cause.^3^, ^6^ Thus, the presence of capillary rarefaction is important because it worsens renal hypoxia and accelerates interstitial fibrosis.^1^ As such capillary rarefaction might be a key target for innovative therapies.^1^, ^2^ CKD in cats is characterized by tubulointerstitial inflammation and fibrosis, tubular atrophy, and secondary glomerulosclerosis.^7^, ^8^ The presence and degree of capillary rarefaction in cats with CKD have not been well defined. In a single published report of peritubular capillary density in a small number of cats there was no evidence of capillary rarefaction.^9^ For novel therapies targeting capillary rarefaction to be relevant in cats with CKD, it must be demonstrated that the pathophysiologic mechanisms are similar to those described in humans. Therefore, the aim of this study was to characterize peritubular capillary density in cats with CKD with the aid of CD31 immunohistochemistry.

We hypothesized that cats with CKD will have a higher degree of capillary rarefaction (represented by a decrease in capillary size and percent capillary area) in comparison to unaffected senior and adult cats; and cats with more advanced CKD (International Renal Interest Society [IRIS] Stage 4) will have a greater degree of capillary rarefaction than cats with IRIS Stage 2 and 3 CKD. A secondary aim was to evaluate the correlation between histological scores of fibrosis, inflammation and glomerulosclerosis, and capillary rarefaction.

## 2 MATERIALS AND METHODS

### 2.1 Study design and selection of animals

The study was performed using archived tissues from necropsy, and consent for necropsy was obtained from the owner of the institution at the time of death. Archived feline necropsy cases with available tissue, serum biochemistry, and urinalysis were found using patient database searches at Colorado State University, The Ohio State University, North Carolina State University, and Royal Veterinary College. Inclusion criteria for CKD cats included serum creatinine concentration >1.6 mg/dL (reference range 0.8‐2.4 mg/dL) associated with a urine specific gravity (USG) <1.035, as well as a clinical diagnosis of CKD based on a combination of physical examination findings, persistent renal azotemia (on at least 2 occasions), and ultrasonographic or radiologic changes consistent with CKD. CKD cats were staged and substaged based on IRIS guidelines for serum creatinine concentration, systemic hypertension, and proteinuria (http://www.iris-kidney.com/guidelines/staging.html). Hypertensive status was defined as follows: nonhypertensive: documentation of blood pressure <150 mmHg; hypertensive: substaged as such by attending clinician, receiving amlodipine treatment, or documented evidence of target organ damage. Cats for whom substage determination was not possible because of uninterpretable results or lack of data were excluded. Additional exclusion criteria for CKD cats included diabetes mellitus, hyperthyroidism, renal neoplasia, polycystic kidney disease, pyelonephritis or urinary tract infection, ureteral obstruction (defined based on a variable combination of medical record review, imaging when available, and necropsy findings), feline infectious peritonitis, feline leukemia virus infection, feline immunodeficiency virus infection, NSAID administration, and chemotherapy (recorded history of administration within 2 months of euthanasia). Inclusion criteria for unaffected cats included serum creatinine concentration <1.6 mg/dL and USG > 1.035. Exclusion criteria for unaffected cats included the same abnormalities listed previously for cats with CKD, and additionally hematuria and proteinuria. Adult cats were defined as those 1 to 10 years of age and senior cats were defined as those 10 years of age and older.

### 2.2 Immunohistochemistry

Formalin‐fixed paraffin‐embedded kidney tissues were sectioned at 3 μm and randomized into 4 runs with equal distribution of unaffected adult, unaffected senior, and CKD samples for immunohistochemistry. Two control slides from the same 2 cats (CKD positive and unaffected adult negative) were included in each run. The slides were deparaffinized and rehydrated with a graded ethanol series. Heat‐induced epitope retrieval was performed by decloaking chamber with citrate buffer (51699; Dako; Carpinteria, California). The slides were then rinsed with distilled water and in methanol hydrogen peroxide solution followed by wash buffer (53006; Dako; Carpinteria, California). Slides were further processed by a Dako Autostainer (S3400, Agilent, Santa Clara, California). Materials used were: antibody diluent with background reducing components (53022; Dako; Carpinteria, California), protein block serum‐free ready to use (X0909; Agilent Technologies; Santa Clara, California), liquid DAB+ Substrate chromogen system (K3468; Dako; Carpinteria, California), rabbit on rodent HRP‐Polymer (RMR622H; Biocare Medical; Pacheco, California), anti‐CD31 antibody (28 364; Abcam; Cambridge, UK) at 1:100 dilution, and FLEX negative control rabbit immunoglobulin (isotype control). Once the slides completed the program in the autostainer, they were counterstained with hematoxylin, dehydrated, and mounted in xylene‐based mounting medium.

### 2.3 Capillary assessment

**FIGURE 1:**
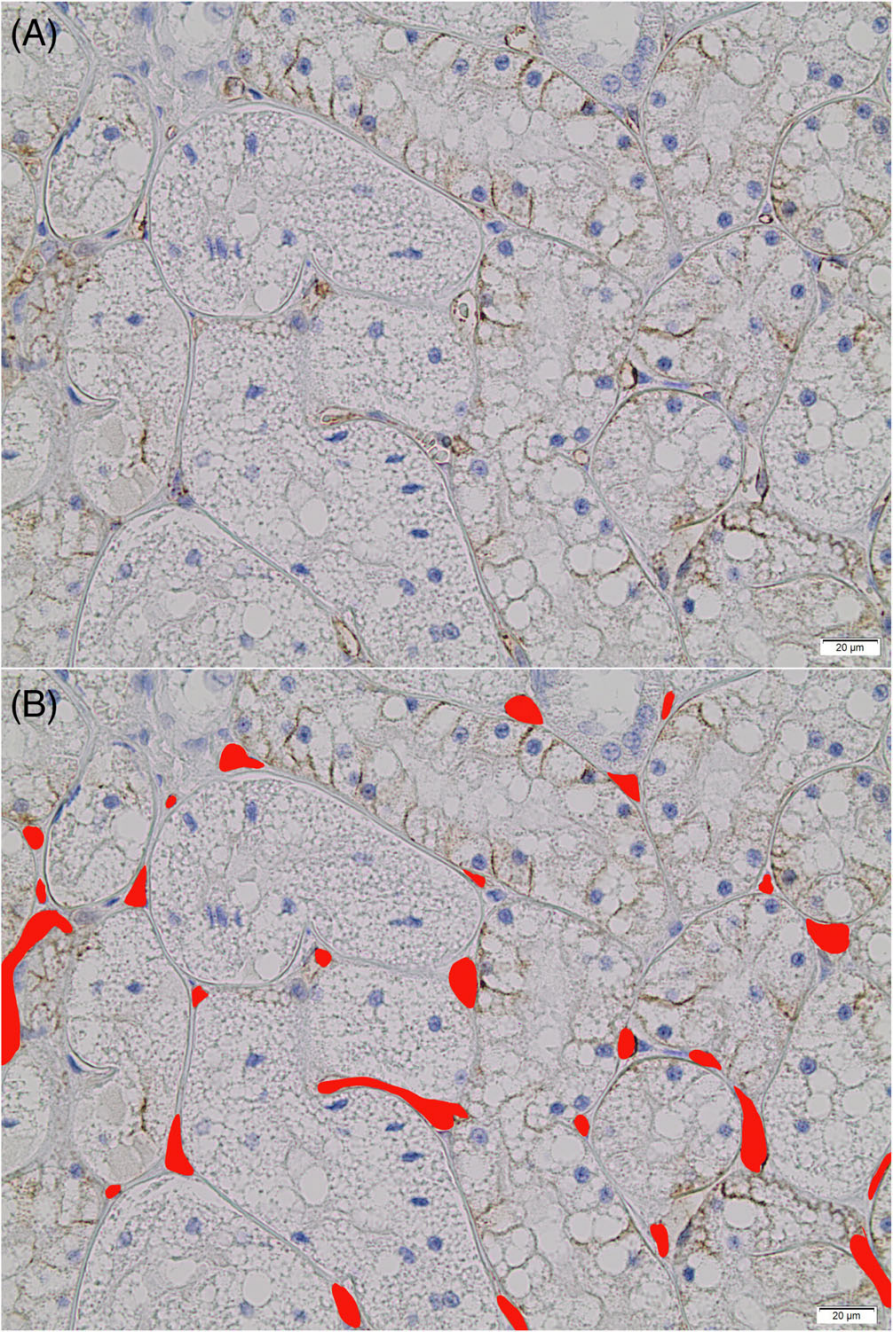
(A) Example cortex image from a normal adult taken by CellSense Standard Program at ×40 magnification that was hand traced and colored by Adobe Photoshop (B). The colored capillaries were counted with Image J cell counter notice plugin and the area calculated by Image J color threshold selection and measurement tool. Scale bar represents 20 μm

The slide evaluator (RP) was trained by a board‐certified pathologist (RC) to identify peritubular capillaries by visual assessment, therefore the CD31 staining was an aid in identification, but not solely relied on. The slides were labeled a random number (1‐78) so the reader was masked to which group the kidney sections belonged and was unbiased during assessment. The cortex and CMJ were delineated. Then representative fields were digitally photographed, with the intention of systematic but randomized selection of fields along the entire region; based on tissue available. Cell Sens Standard Program was used to take 10 cortex pictures and 5 CMJ pictures at ×40 objective for each sample. Glomeruli and large arterioles were avoided in the images taken and not included in capillary counts or area. Medulla also was not scored as the amount harvested in each slide was highly variable. Adobe Photoshop Sketch and Adobe Photoshop 2020 were used to hand trace and color the area of the capillaries red. Once colored, the images were uploaded to Image J. The programs used were “plug‐in cell counter notice” to count capillaries and “color threshold and analyze” to calculate the area in pixels of the red‐colored sections of capillaries. Figure 1 shows an example of before and after colored capillaries.

The number of capillaries per image and total capillary area (total red pixels on 1 image) for both cortex and CMJ were determined. In addition, average capillary size (defined as the total area of capillaries divided by the number of capillaries per image), and average percent capillary area (defined as the total area of capillaries divided by the total area of the image), were calculated for each sample. The  variables capillary count, capillary size, and percent capillary area were used as an assessment of capillary rarefaction.

### 2.4 Histologic scoring

Formalin‐fixed paraffin‐embedded kidney tissues were sectioned at 3 μm and stained with routine hematoxylin and eosin (H&E) and Masson's trichrome stain. Masson's trichrome stain was utilized to assess glomerulosclerosis and interstitial fibrosis. Histologic scoring was performed by a pathologist masked to clinical data (RC). Fifty randomly selected glomeruli per case were examined. If 2 sections of the same kidney were available for evaluation, then 25 glomeruli per section were examined. The number of globally sclerotic glomeruli characterized by hypo‐ to acellular meshworks of collagen were counted and the overall global glomerulosclerosis percentage was calculated. To assess fibrosis and inflammation, 10 randomly selected fields (×40) from both the cortex and the corticomedullary junction were examined. Each field was assigned a score for the severity of the interstitial fibrosis (by Masson's trichrome) and a score for inflammation (by H&E) as follows: 0 = not present; 1 = lesion present without disruption of the tubular architecture; 2 = lesion widely separates tubules; 3 = lesion replaces tubules (scoring system used for both fibrosis and inflammation). The mean score for the 10 fields was calculated for both fibrosis and inflammation in both the cortex and the corticomedullary junction regions of the kidney.

### 2.5 Statistical analysis

All datasets were tested for normality by D'Agostino and Pearson test and statistical analysis were carried out in Prism software (Prism 9, Prism Graphpad IN., La Jolla, California). A value of *P* < .05 was considered significant for all analyses. Mann‐Whitney analysis was used for the following comparisons: capillary count between cortex and CMJ, capillary count, and capillary size in the cortex and CMJ between unaffected cats vs CKD cats, unaffected senior cats vs CKD cats, unaffected adult cats vs CKD cats, and between hypertensive vs nonhypertensive CKD cats. A *t* test was used to compare percent capillary area in the cortex and CMJ between unaffected cats vs CKD cats, unaffected senior cats vs CKD cats, and unaffected adult cats vs CKD cats. A Kruskal‐Wallis test with Dunn's post hoc analysis (adjusted p values provided by prism for post hoc analysis) was used for the following comparisons: capillary size in the cortex and CMJ between unaffected cats vs CKD IRIS stage 2 and 3 vs IRIS stage 4 cats, capillary size and percent capillary area in the cortex and CMJ between CKD IRIS stage 2 vs IRIS stage 3 vs IRIS stage 4 cats, and between proteinuric, borderline proteinuric, and nonproteinuric CKD cats. An ordinary 1‐way ANOVA with Dunn's post hoc analysis was used to compare percent capillary area in the cortex and CMJ between unaffected cats vs CKD IRIS stage 2 and 3 vs IRIS stage 4 cats. Spearman rank was used to assess the correlation between serum creatinine concentration and capillary size and percent capillary area in the cortex and CMJ as well as between histopathologic scores (percent global glomerulosclerosis, fibrosis, and inflammation) and capillary size and percent capillary area, and age and capillary size and percent capillary area from both regions in unaffected cats.

## 3 RESULTS

### 3.1 Animals

Tissues from 58 cats with CKD (16 IRIS Stage 2 CKD, 18 IRIS Stage 3 CKD, and 24 IRIS Stage 4 CKD), 10 unaffected adult and 10 unaffected senior cats were utilized in the study. The CKD group consisted of 37 domestic short hairs (DSH), 9 domestic long hairs (DLH), 7 Siamese, 2 Persians, 1 Russian blue, 1 Tonkinese, and 1 Maine Coon; 37 male neutered and 21 female spayed. The unaffected senior group included 8 DSH, 1 Persian, and 1 British Shorthair; 9 male neutered and 1 female spayed. The unaffected adult group consisted of 7 DSH, 1 DLH, 1 Egyptian Mau, and 1 Bengal; 5 male neutered and 5 female spayed. One cat in the adult group was initially categorized as 1 year of age, but on further investigation after analysis was found to be less than 1 year of age. For simplicity, this single cat was still included in the terminology “adult.” Causes of death/euthanasia in the unaffected group were neoplasia not affecting the kidney (8), respiratory disease (3), undetermined (4), neurologic disease (3), liver disease (1), and research cat sacrificed for unrelated study (1). Signalment comparisons between the 3 groups are presented in Table 1. When CKD cats were compared to all unaffected cats, CKD cats were significantly older than all unaffected cats (*P* = <.001). Serum creatinine concentration, systemic hypertension status, proteinuria status, and histological scoring for each group are also presented in Table 1.

**TABLE 1:** Age, serum creatinine concentration, systemic hypertension status, proteinuria status, and histological scoring for each group

	Unaffected Adult Cats (n = 10)	Unaffected Senior Cats (n = 10)	All Unaffected Cats (n = 20)	Cats with CKD (n = 58)
Age (years)^a^ ^,^ ^c^	5 (0.6‐9)	14 (10‐16)	9.5 (0.6‐16)	15 (3‐21)
Creatinine (range in mg/dL)^a^ ^,^ ^b^ ^,^ ^c^	1.1 (0.7‐1.5)	1.1 (0.5‐1.5)	1.0 (0.5‐1.5)	4.3 (1.6‐13.6)
Systemic hypertension	NA	NA	NA	32 non hypertensive; 26 hypertensive
Proteinuria	NA	NA	NA	16 nonproteinuric; 17 borderline proteinuric; 23 proteinuric
% Global glomerulosclerosis^a^ ^,^ ^c^	0 (0‐16)	3 (0‐48)	2 (0‐48)	20 (0‐92)
Fibrosis score cortex^a^ ^,^ ^b^ ^,^ ^c^	0 (0‐0.8)	0.2 (0‐0.6)	0.05 (0‐0.8)	0.7 (0‐2.7)
Inflammatory infiltrate score cortex^a^ ^,^ ^b^ ^,^ ^c^	0 (0‐0.9)	0.2 (0‐0.7)	0 (0‐0.9)	1 (0‐2.9)
Fibrosis score CMJ^a^ ^,^ ^b^ ^,^ ^c^	0 (0‐0.4)	0.1 (0‐1)	0 (0‐1)	1.5 (0.1‐2.5)
Inflammatory infiltrate score CMJ^a^ ^,^ ^b^ ^,^ ^c^	0 (0‐0.4)	0.05 (0‐0.5)	0 (0‐0.5)	1.3 (0‐2.5)

*Note*: Data presented as median (range).

Abbreviations: CKD, chronic kidney disease; CMJ, corticomedullary junction; NA, not available.

^a^ Significant difference between all unaffected cats and CKD.

^b^ Significant difference between unaffected senior and CKD.

^c^ Significant difference between unaffected adult and CKD.

### 3.2 Capillary count

**FIGURE 2:**
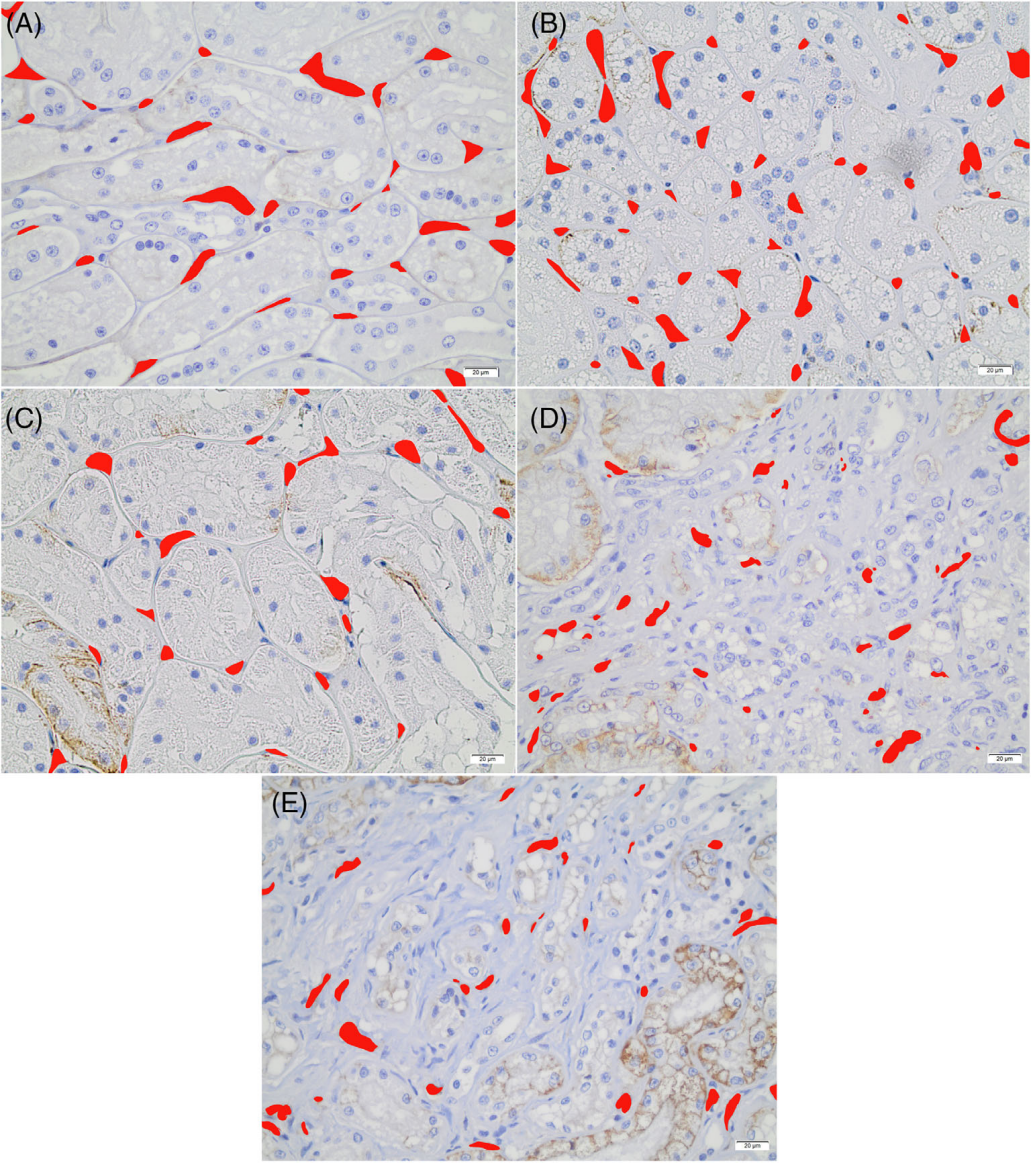
Example of colored capillary images in the renal cortex at ×40 magnification from each group (A) unaffected adult, (B) unaffected senior, (C) IRIS Stage 2, (D) IRIS Stage 3, and (E) IRIS Stage 4. Scale bar represents 20 μm

Figure 2 illustrates example images of capillary structures identified and colored for analysis in the renal cortex from each group. In both CKD cats and unaffected cats, capillary count was significantly higher in the CMJ compared to the cortex (*P* = <.001 and *P* = .0015, respectively). The number of capillaries per image was not significantly different between unaffected and CKD cats in either the cortex or CMJ (*P* = .29 and *P* = .08, respectively).

### 3.3 Capillary size and percent capillary area

**FIGURE 3:**
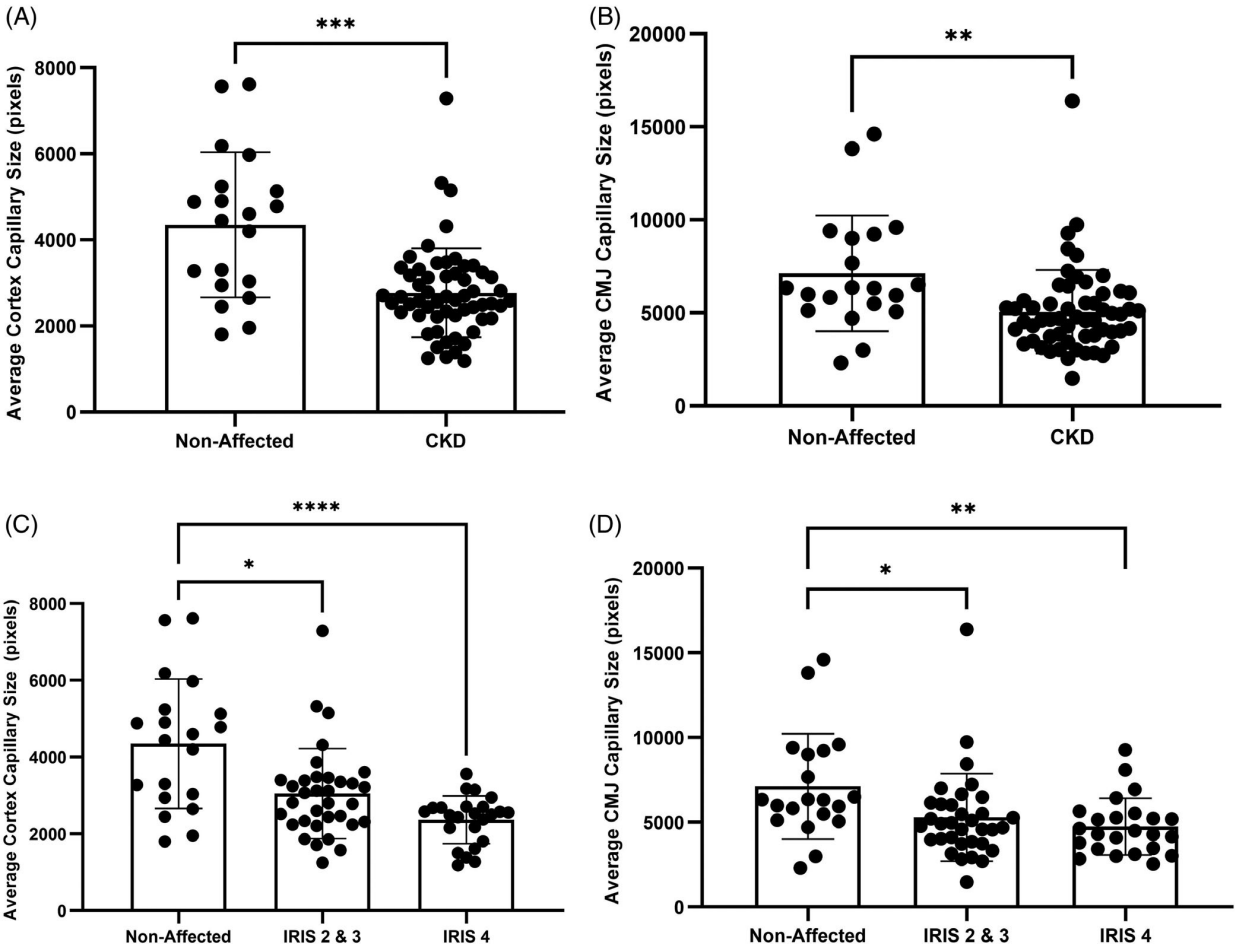
Capillary size was significantly lower in cats with chronic kidney disease (CKD) in comparison to unaffected cats in (A) renal cortex (*P* = <.001; Mann‐Whitney test) and (B) the renal CMJ (*P* = .001; Mann‐Whitney test). International Renal Interest Society (IRIS) stage 2/3 cats and IRIS Stage 4 cats had significantly lower capillary size in comparison to unaffected cats in (C) the renal cortex (*P* = .03 and *P* = <.001, respectively; Kruskal‐Wallis test), and (D) the renal CMJ (*P* = .03 and *P* = .006 respectively; Kruskal‐Wallis test). There was no significant difference in capillary size between IRIS stages 2/3 and 4 in either region

**FIGURE 4:**
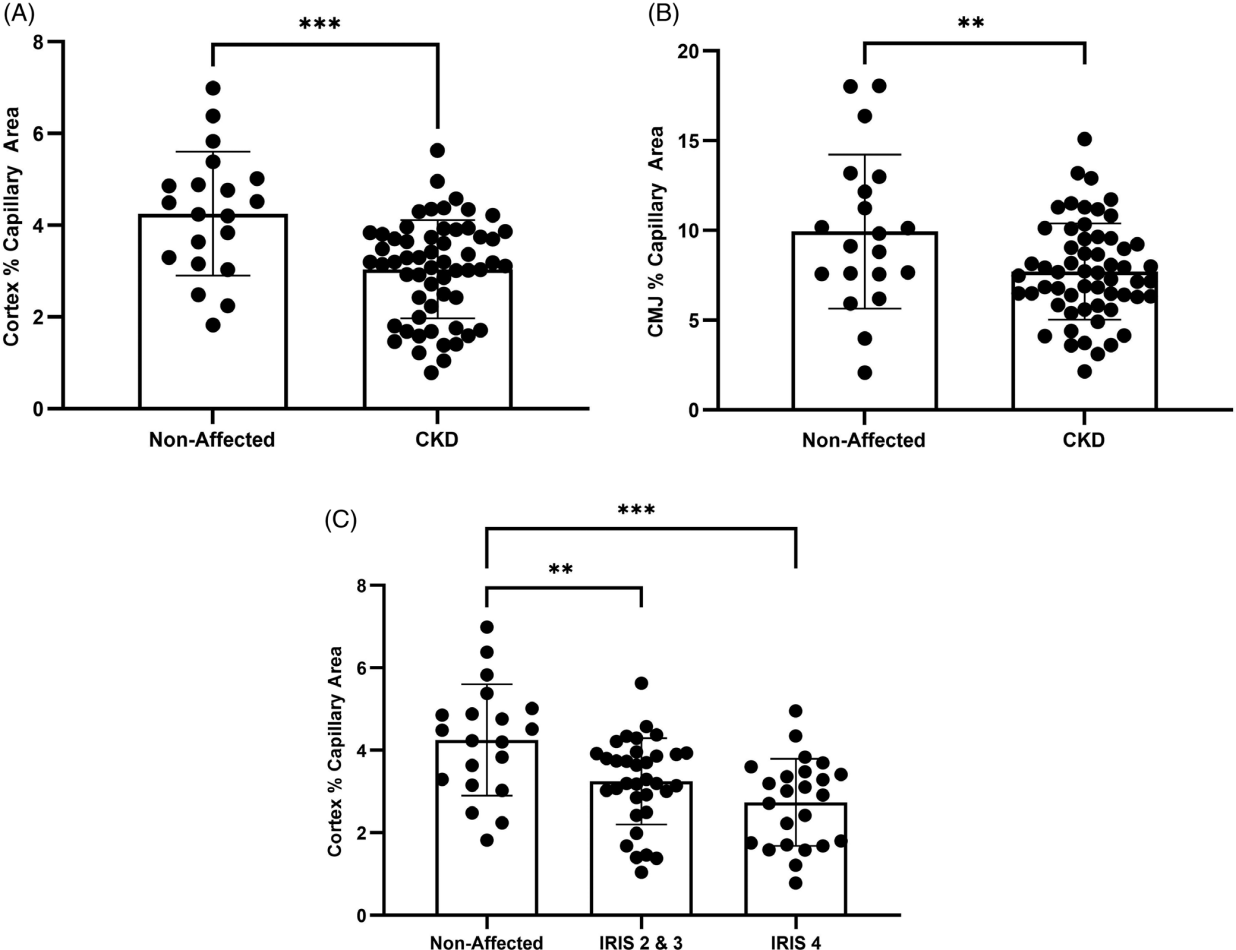
Percent capillary area was significantly lower in cats with chronic kidney disease (CKD) in comparison to unaffected cats in (A) the renal cortex (*P* = <.001; *t* test) and (B) the renal CMJ (*P* = .008; *t* test). (C) International Renal Interest Society (IRIS) stage 2/3 cats and IRIS Stage 4 cats had significantly lower percent capillary area in comparison to unaffected cats (*P* = .007 and *P* = <.001, respectively; Ordinary 1‐way ANOVA). There was no significant difference in percent capillary area between IRIS stages 2/3 and 4

Data for capillary size and percent capillary area for each group are displayed in Table 2. Capillary size (Figure 3) and percent capillary area (Figure 4) were significantly lower in cats with CKD in comparison to unaffected cats in both the cortex and CMJ. No significant difference in capillary size or percent capillary area was found between CKD cats of different IRIS stages in the cortex (*P* = .054 and *P* = .12, respectively) or CMJ (*P* = .73 and *P* = .67, respectively).

**TABLE 2:** Capillary count, size, and percent capillary area in cortex and corticomedullary junction of unaffected cats and cats with chronic kidney disease

	All Unaffected Cats (n = 20)	Unaffected Adult Cats (n = 10)	Unaffected Senior Cats (n = 10)	All CKD Cats (n = 58)	IRIS Stage 2 & 3 CKD Cats (n = 34)	IRIS Stage 4 CKD Cats (n = 24)
Cortex						
Capillary count/image	31.5 (18.5‐51.8)	31.2 (18.9‐46.8)	31.6 (18.5‐51.8)	34.7 (10.7‐90.6)	34.1 (17.6‐50)	35.8 (10.7‐90.6)
Capillary size (pixels)^a^ ^,^ ^b^ ^,^ ^c^ ^,^ ^d^ ^,^ ^e^	4523 (1801–7618)	4322 (1960‐7573)	4744 (1801‐7618)	2591 (1184‐7289)	2944 (1247‐7289)	2516 (1184‐3562)
Percent capillary area^a^ ^,^ ^b^ ^,^ ^c^ ^,^ ^d^ ^,^ ^e^	4.4 (1.8‐7.0)	4.0 (2.2‐5.4)	4.6 (1.8‐7.0)	3.2 (0.8‐5.6)	3.2 (1.0‐5.6)	3.0 (0.8‐5.0)
CMJ						
Capillary count/image	41.5 (19.6‐120.2)	39.6 (19.6‐120.2)	45.5 (36.8‐64.2)	50.9 (17.4‐88.4)	51.0 (17.4‐78.4)	49.6 (22.8‐88.4)
Capillary size (pixels)^a^ ^,^ ^b^ ^,^ ^d^ ^,^ ^e^	6332 (2302‐14 601)	6416 (2302‐14 601)	6171 (5064‐13 819)	4672 (1477‐16 390)	4850 (1477‐16 390)	4407 (2534‐9269)
Percent capillary area^a^ ^,^ ^b^	9.5 (2.1‐18.1)	8.4 (2.0‐18.0)	10.0 (7.6‐18.1)	7.6 (2.1‐15.1)	7.7 (2.1‐15.1)	7.2 (3.1‐11.7)

*Note*: Data are displayed as median (range).

Abbreviations: CKD, chronic kidney disease; CMJ, corticomedullary junction.

^a^ Significant difference between all unaffected cats and CKD.

^b^ Significant difference between unaffected senior and CKD.

^c^ Significant difference between unaffected adult and CKD.

^d^ Significant difference between unaffected cats and Stage 2/3 CKD cats.

^e^ Significant difference between unaffected cats and Stage 4 CKD cats.

**FIGURE 5:**
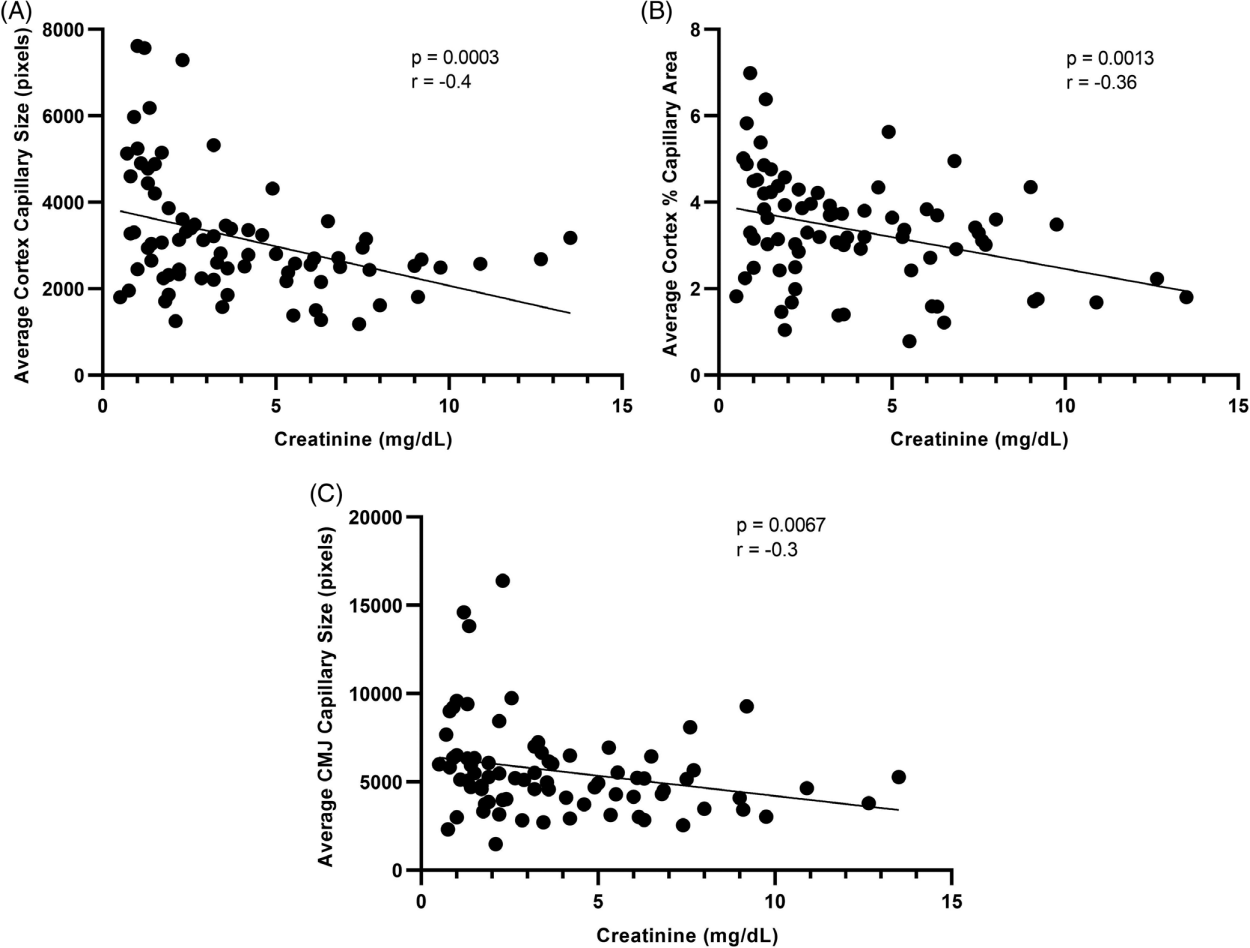
Correlation (Spearman rank) between (A) capillary size in cortex and serum creatinine concentration, (B) percent capillary area in cortex and serum creatinine concentration, and (C) average CMJ capillary size and serum creatinine concentration

Capillary size and percent capillary area in the cortex, and capillary size in the CMJ were negatively correlated with serum creatinine concentration (Figure 5). There was no significant difference in capillary size or percent capillary area between hypertensive and nonhypertensive cats with CKD in the cortex (*P* = .93 and *P* = .25, respectively) or CMJ (*P* = .06 and *P* = .09, respectively). There was no significant difference in capillary size or percent capillary area between nonproteinuric, borderline proteinuric, or proteinuric cats with CKD in the cortex (*P* = .17 and *P* = .70, respectively) or CMJ (*P* = .67 and *P* = .72, respectively).

### 3.4 Capillary rarefaction correlation with histopathology

Table 3 summarizes the histopathologic findings in relation to capillary size and percent capillary area in the cortex and CMJ. Capillary size and percent capillary area were both weakly negatively correlated with percent global glomerulosclerosis, fibrosis, and inflammation in the cortex. In the CMJ, capillary size was weakly negatively correlated with fibrosis and inflammation. Correlation between capillary size and percent global glomerulosclerosis is not relevant in the CMJ because of the limited number of glomeruli in that region.

**TABLE 3:** Correlation between capillary size and percent capillary area and histopathologic scores

	Percent GGS	Inflammatory infiltrate score	Fibrosis score
Cortex			
Capillary size	*r* = −.44	*r* = −.42	*r* = −.38
	*P* = <.001	*P* = <.001	*P* = <.001
Percent capillary area	*r* = −.39	*r* = −.30	r = −.30
	*P* = <.001	*P* = .009	*P* = .007
CMJ			
Capillary size	NA	*r* = −.26	*r* = −.33
		*P* = .023	*P* = .004
Percent capillary area	NA	*r* = −.18	*r* = −.12
		*P* = .11	*P* = .29

Abbreviations: CMJ, corticomedullary junction; GGS, global glomerulosclerosis; NA, not applicable.

## 4 DISCUSSION

The purpose of our study was to determine if capillary rarefaction was a component of CKD in cats. We hypothesized cats with CKD would be more affected by capillary rarefaction than unaffected senior and adult cats. We found average capillary size and percent capillary area were lower in CKD cats in both the cortex and CMJ when compared to unaffected cats. We hypothesized cats with more advanced kidney disease would have a greater degree of capillary rarefaction than earlier‐stage cats, and indeed serum creatinine concentration was correlated with capillary size and percent capillary area in the cortex. Lastly, we hypothesized unaffected senior cats would have an increased degree of capillary rarefaction in comparison to unaffected adult cats as a process of aging from a lifetime of minor insults to the kidney. We did not find a significant difference between unaffected adult and senior cats nor was there a correlation with age. However, based on the variability of the data collected, the study was likely underpowered to assess this secondary aim.

A previous study assessed changes in renal peritubular capillaries in cats with CKD based on CD34 staining and assessment and concluded capillary rarefaction was not a characteristic of cats with CKD.^9^ However, the sample size was small, consisting of 11 cats with CKD and 4 control cats. Details regarding the age and severity of disease were not specified and some CKD cats might have not been azotemic. CD34 is an endothelial cell marker expressed on endothelial cells of both lymphatic and blood vessels and has been used in several studies in humans to aid in the identification of peritubular capillaries.^10^, ^11^ In cats, CD34 and CD31 have both been utilized in the assessment of feline endothelium, specifically vascular neoplasms.^12^, ^13^ CD31 is a transmembrane glycoprotein that is expressed by platelets, megakaryocytes, and endothelial cells and was found to have less background staining than CD34 in feline vascular neoplasms.^13^ This made CD31 a good potential candidate for identifying capillaries to evaluate peritubular capillary density in the feline kidney as it also has been used to assess capillary rarefaction.^2^ Initial optimization studies in our lab (unpublished data) concurred with previously published data and indicated that CD31 would perform better than CD34 for our aims, especially when combined with visual assessment of each slide by a trained investigator.^13^ Techniques for assessing and quantifying capillary structures also differed between Nakamura et al. and the present investigation and therefore the studies are not directly comparable.^9^ The technique used in the previous investigation most closely aligned with the capillary count as described in the current study.

A significant difference in capillary count between unaffected and CKD cats was not seen in the present study, but has been demonstrated in human CKD patients.^14^ One explanation is the endothelial cells that are nonfunctional might still stain with CD31 even though they line atrophic or stenotic capillaries. Therefore, count might be similar across the groups but the percent capillary area might still be different. Another explanation for not finding an effect on capillary count in cats could be unknown species differences such as pathophysiology or differing chronicity of disease, or therapeutic approaches. For example, there is evidence that inhibition of the renin‐angiotensin system (RAS) is protective against capillary rarefaction.^15^ However RAS inhibition is not a routine clinical recommendation in cats with CKD given that in this species it is less likely associated with proteinuria than canine CKD.^16^ In the present study it was not possible to document which cats might have had consistent administration of anti‐hypertensive medications or RAS inhibitors because of the retrospective nature of the study and the difficulty medicating the cats. Therefore, a conclusion about the effects of these medications on capillary rarefaction in this population of cats could not be made.

Peritubular capillary rarefaction has been extensively studied in human literature and is identified in both acute and chronic kidney diseases as well as experimental animal models of disease.^1^ It has been seen in diabetic nephropathy, hypertensive kidney disease, IgA nephropathy, congenital nephrotic syndrome, lupus nephritis, polycystic kidney disease, unilateral ureteral obstruction, remnant kidney model, glomerulonephritis, and allograft nephropathy in humans.^1^, ^2^ Several of these processes cause CKD in humans, supporting the hypothesis that capillary rarefaction contributes to the development of CKD. In addition, capillary rarefaction is observed before the onset of overt fibrosis, suggesting it is an early sign of renal dysfunction.^1^

Interstitial fibrosis is the renal lesion that best correlates with severity of azotemia, hyperphosphatemia, and anemia.^7^ A significant positive correlation between capillary rarefaction and fibrosis was observed in this study. Similar findings occur in human kidney disease where there is a negative correlation between capillary density and the severity of fibrosis.^2^, ^14^ Mechanistically, there is likely a close relationship between capillary damage and the formation of fibrosis. After kidney injury, pericytes migrate away from peritubular capillaries and then differentiate into 2 scar‐forming cell types (myofibroblasts and activated fibroblasts).^2^ Pericyte loss potentiates further tubular damage, and injured tubules increase transforming growth factor (TGF‐B1) expression, which promotes endothelial cell apoptosis and pericyte to myofibroblast differentiation.^1^, ^2^ Collectively these findings support the association of capillary rarefaction with disease severity in cats with CKD identifying this process as a potential driver of disease progression.

There were no significant differences in the degree of capillary rarefaction in cats with proteinuria or systemic hypertension. It is possible that variability in the duration of time that the patient was affected (diagnosed shortly before death vs managed for several years) could have affected these results, or that the study was underpowered to appreciate these differences. Proteinuric conditions are thought to lead to the release of inflammatory cytokines from tubular cells which can instigate capillary rarefaction in humans.^1^ In addition, systemic hypertension exacerbates intraglomerular hypertension, damaging glomeruli, and leading to sclerosis and subsequent loss of vasculature. In humans, hypertensive kidney disease is 1 of the major causes of CKD and several anti‐hypertensive drugs decrease peritubular capillary rarefaction in experimental animal models.^2^, ^15^, ^17^ The cause and effect relationship between systemic hypertension and CKD in cats is not yet fully elucidated.

Vascular endothelial growth factor (VEGF) preserves capillary endothelium and partially reverse impaired angiogenesis.^3^ In fact, loss of tubular epithelial VEGF induces peritubular capillary rarefaction even without kidney injury, and conversely exogenous VEGF in some kidney disease models maintains peritubular capillaries, restore renal function, and ameliorate tubulointerstitial fibrosis.^2^, ^6^ In cats with CKD, renal transcript levels of VEGF are lower than control cats and are negatively associated with histologic scores.^18^ Urinary cytokine concentrations of VEGF are also significantly lower in CKD cats vs normal cats.^19^ Thus, there is likely a deficiency in the kidney's ability to maintain vascular health through this mechanism. VEGF could be considered a potential therapeutic target for capillary rarefaction, but overexpression of VEGF is detrimental^6^; therefore, achieving an appropriate balance might be challenging. Hypoxia‐inducible factor (HIF), a master gene regulator of cellular response to hypoxia that upregulates more than 100 genes such as erythropoietin and VEGF is another potential therapeutic target to address capillary rarefaction.^2^, ^4^, ^20^ One study evaluating HIF‐1α gene transcription (12 cats with naturally occurring CKD and 8 healthy control cats) found higher transcription levels of HIF‐1α in the kidney of CKD cats than in the control cats suggesting chronic hypoxia as a feature of disease.^18^ In a second study of experimentally induced ischemic CKD, transcription levels of HIF‐1α were positively correlated with worsening fibrosis scores.^21^ These findings suggest that addressing hypoxia through these mechanisms could be a therapeutic target for cats with CKD.^4^ For example, HIF stabilizers such as prolyl hydroxylase inhibitors (eg, roxadustat) might improve capillary rarefaction by both addressing anemia and supporting vascular health.^2^, ^4^, ^20^

Limitations of this study included selection bias at necropsy whereupon a pathologist might have chosen a section that was grossly abnormal for evaluation. Thus, it is possible that archived biopsy sections were not always representative of the cat's entire kidney. Patients with CKD are more likely to have decreased kidney size in comparison to unaffected cats. Information on total kidney size at necropsy/biopsy was not available, therefore total volume of kidney could not be incorporated into the measurements of capillary size or percent capillary area. Instead, a consistent number of ×40 fields were evaluated to compare between groups. Some of the blocks that the slides were cut from were older and thus antigen retrieval could have been affected. Orientation of embedding and cutting could have variably affected the appearance of capillary size and shape. Recent work has demonstrated the gold standard for assessing peritubular capillary density is microangio‐CT of the entire kidney.^22^ Histologic measurements overestimate capillary density because of the artifacts from sample sectioning, mounting, and such. Such analysis was not possible in the archived tissues used in our study. However, the slides used in the study were processed according to routine histopathology guidelines, therefore possible changes to capillary size would have been similar throughout the sample set. Variability in endothelial cell staining as well as the presence of background staining was compensated for by individual assessment of each slide by a reviewer who was trained to identify peritubular capillaries. This was also a challenge of the study; tracing, coloring, and counting capillaries of 1170 images was a time‐consuming process, however, the individual assessment of each image by the investigator and overseeing pathologist should enhance the reliability of the results.

Additional limitations of this study include its retrospective nature, which could have influenced the reliability of the clinical data. Although all available medical records and historical clinicopathologic data for each case were utilized to determine IRIS stage and substage, data were sometimes not available at the time of death, so a cut‐off of 2 months was used for inclusion. For the unaffected cat group, not enough blood pressure data were available from the medical records to allow for analysis, and UPC data was not available at all (however proteinuria on urinalysis was an exclusion criteria).

In conclusion, demonstrating the existence of capillary rarefaction in cats with CKD enhances our understanding of disease pathophysiology and is consistent with findings in other species. Capillary rarefaction might prove to be an important mediator of progression of disease in cats and represents a novel therapeutic target. Interventional studies are warranted to determine whether this is indeed the case.

## CONFLICT OF INTEREST DECLARATION

Dr Quimby is on the Advisory board for Nestle Purina PetCare; speaker honoraria Dechra, Nestle Purina Petcare, Heska; consulting, Dechra, BI, Elanco, Vetoquinol, Kidney‐Chek; travel covered Nestle. Research funding from Nestle Purina Petcare, Triviumvet, member of the International Renal Interest Society Board. Dr Elliott received funding from Consultancies: Elanco Ltd, CEVA Animal Health Ltd, Boehringer Ingelheim Ltd, MSD Animal Health Ltd., Orion Incorp, Idexx Ltd, Nextvet Ltd, Waltham Petcare Science Institute, Kindred Biosciences Inc, Invetx Inc; Zoetis Ltd, and grand funding from Elanco Ltd, Waltham Petcare Science Institute, Royal Canin SAS, Idexx Ltd., CEVA Animal Health, Member of the International Renal Interest Society Board. Dr Cianciolo was a full‐time The Ohio State University employee at the time the study was conducted, she now works 75% time for Zoetis. Drs Paschall, Lunn, and McLeland declare no conflict of interest.

## OFF‐LABEL ANTIMICROBIAL DECLARATION

Authors declare no off‐label use of antimicrobials.

## INSTITUTIONAL ANIMAL CARE AND USE COMMITTEE (IACUC) OR OTHER APPROVAL DECLARATION

Authors declare no IACUC or other approval was needed.

## HUMAN ETHICS APPROVAL DECLARATION

Authors declare human ethics approval was not needed for this study.
